# Nitrogen Fertilisation Increases Specific Root Respiration in Ectomycorrhizal but Not in Arbuscular Mycorrhizal Plants: A Meta-Analysis

**DOI:** 10.3389/fpls.2021.711720

**Published:** 2021-08-06

**Authors:** Bahareh Bicharanloo, Timothy R. Cavagnaro, Claudia Keitel, Feike A. Dijkstra

**Affiliations:** ^1^School of Life and Environmental Sciences, Sydney Institute of Agriculture, The University of Sydney, Camden, NSW, Australia; ^2^School of Agriculture, Food and Wine, The University of Adelaide, Adelaide, SA, Australia

**Keywords:** association, carbon cost, expenditure, meta-regression, multi-level, multi-model inference, symbiosis, uptake

## Abstract

Plants spend a high proportion of their photosynthetically fixed carbon (C) belowground to support mycorrhizal associations in return for nutrients, but this C expenditure may decrease with increased soil nutrient availability. In this study, we assessed how the effects of nitrogen (N) fertiliser on specific root respiration (SRR) varied among mycorrhizal type (Myco type). We conducted a multi-level meta-analysis across 1,600 observations from 32 publications. SRR increased in ectomycorrhizal (ECM) plants with more than 100 kg N ha^−1^ applied, did not change in arbuscular mycorrhizal (AM) and non-mycorrhizal (NM) plants, but increased in plants with a dual mycorrhizal association in response to N fertilisation. Our results suggest that high N availability (>100 kg N ha^−1^) could disadvantage the growth of ECM plants because of increased C costs associated with maintaining higher root N concentrations, while the insensitivity in SRR by AM plants to N fertilisation may be because AM fungi are more important for phosphorus (P) uptake.

## Introduction

Plants allocate up to 50% of photosynthetically fixed carbon (C) to root biomass and rhizodeposition (Pausch and Kuzyakov, 2018). In addition, plants spend a significant portion of fixed C to support mycorrhizal fungi in exchange for soil-derived nutrients (Smith et al., 2009); most of this C expenditure is associated with root and hyphal respiration (Hughes et al., 2008). Up to 86% of all flowering plants can form symbiotic associations with mycorrhizal fungi (Brundrett, 2009; van der Heijden et al., 2015), where associations with arbuscular mycorrhizal (AM) and ectomycorrhizal (ECM) fungi are the most widespread type of mycorrhiza (Brundrett, 2009). The C cost associated with forming and maintaining different types of mycorrhizas remains highly uncertain, with estimates as low as 4% and as high as 25% of the net primary production of a plant allocated to the fungi (Hobbie, 2006; Johnson and Gehring, 2007; Stuart and Plett, 2020). Plants associated with AM and ECM fungi differ in their nutrient economies and related biogeochemical transformations of C and nutrients (Phillips et al., 2013). Thus, it can, therefore, be expected that variation in nutrient availability will also affect root respiratory C costs of AM and ECM plants, with consequences for their productivity and abundance in terrestrial ecosystems.

In general, the mycorrhizal contribution to plant nitrogen (N) acquisition is higher for ECM than for AM fungi, while AM fungi tend to have a more important role in phosphorus (P) uptake than ECM fungi (van der Heijden et al., 2015). Whereas, ECM plants also tend to dominate in organic soils with relatively high C:N ratios (Averill et al., 2014; Jo et al., 2019; Soudzilovskaia et al., 2019), AM plants tend to dominate in mineral soils with lower C:N ratios and higher net N mineralisation rates (Johnson, 2010; Lin et al., 2017; Jo et al., 2019). ECM plants have an advantage in organic soils, because ECM fungi are able to decompose soil organic matter and acquire organic N *via* the production of specialised enzymes (Hobbie et al., 2013). However, because the production of these C enzymes is expensive (Soudzilovskaia et al., 2015), ECM symbiosis of C may also be costly to the plant under these conditions. Unlike ECM, AM fungi have no specialised enzymes for organic N acquisition (Terrer et al., 2016; Jansa et al., 2019) and are not able to decompose soil organic matter (SOM) directly, but they can accelerate the decomposition indirectly by translocating plant-derived C to soil microorganisms to stimulate their activity (Hodge and Fitter, 2010; Taylor et al., 2016).

Nitrogen availability can affect the plant-fungi relationship by changing the balance between costs and benefits of forming mycorrhizal associations (Phillips et al., 2013). Reliance of plants on mycorrhizal symbiosis reduces when N availability increases and is more easily accessible to plants; therefore, plants may reduce their C investment to mycorrhizas with increased N fertilisation (Högberg et al., 2010; Thirkell et al., 2019). N fertilisation further can induce P limitation, which could shift C expenditure from acquiring N to acquiring P (Johnson, 2010; Treseder et al., 2018). The effects of N availability on mycorrhizas have mostly been assessed through changes in colonisation percentage per length of root (for AM) or root tips (for ECM), showing an overall decrease of 15% with N fertilisation across different studies, in a meta-analysis (Treseder, 2004), but there is large variation among studies. Indeed, AM colonisation could increase with N fertilisation because AM fungi also have a substantial N demand, thereby competing with plants for N (Puschel et al., 2016; Wang et al., 2018), or because of a shift towards increased plant P demand (Corkidi et al., 2002).

Given that N fertilisation affects mycorrhizal colonisation, this should result in changes in C costs for plants to maintain mycorrhizal associations. Indeed, specific root respiration (SRR) rate, which is defined as the root respiration rate per unit of root biomass (Pregitzer et al., 2007), tends to be greater for roots in association with mycorrhizal fungi, because mycorrhizal respiration also contributes to SRR (Hughes et al., 2008). SRR can be directly measured on excised roots (Makita et al., 2012; Wang and Liu, 2014), which in most cases severs external hyphae, and, therefore, most likely only includes the contribution of intraradical hyphae (for AM) and other mycorrhizal structures in or on the surface of roots (for AM and ECM). When root respiration is indirectly measured as the difference in soil respiration rates between soils with and without live roots present (Ding et al., 2010; Tu et al., 2013), it not only includes respiration of external hyphae but also respiration from recent rhizodeposits.

In theory, increased N availability with N fertilisation reduces plant reliance on mycorrhizal symbiosis, which may result in a decline of mycorrhizal colonisation and, thence, a lower SRR rate. However, evidence for this is sparse; most N fertilisation studies have concentrated on examining changes in root respiration and have not accounted for concurrent changes in root biomass. For instance, root respiration in an ECM boreal forest increased at a low level of N fertilisation (20 kg N ha^−1^) but decreased at a high level of N (100 kg N ha^−1^) (Hasselquist et al., 2012), while chronic N fertilisation had no effect on root respiration in northern hardwood forests dominated by AM trees (Burton et al., 2004). It is difficult to pinpoint the cause of the variability in responses in root respiration, which could be due to the level of N fertiliser applied and due to the variable N fertilisation effects on root biomass (so that fertilisation effects on root respiration may not be the same as SRR), on mycorrhizal colonisation rates of ECM and AM, and/or on root N concentrations. Indeed, increased root N concentrations are linked to higher SRR rates (Reich et al., 2008), but increased N in roots can be a result of either direct root uptake of N or mycorrhizal support.

While mycorrhizas represent a significant sink for plant photoassimilates, and the amount of C invested is likely to differ with Myco type and N supply, the impacts of Myco type and N fertilisation on SRR are less clear. Therefore, our meta-analysis included an investigation of the effects of N fertilisation on SRR to assess N fertiliser effects on C loss per unit of root biomass for plants associated with different types of mycorrhizas. The effects of N fertilisation on soil C fluxes and their components (heterotrophic and autotrophic respiration) have been reviewed (Zhou et al., 2014), but to our knowledge, this is the first study synthesising N fertilisation effects on SRR by plants varying in mycorrhizal symbiosis type through a meta-analysis. We focused on SRR (i.e., respiration per unit of root biomass) rather than total root respiration as a measure of plant C costs because it accounts for potential confounding effects of N fertilisation on root biomass. Environmental conditions such as soil moisture (Soil moist) and temperature can also affect SRR mediated by N fertilisation (Zhang et al., 2014). We, therefore, used multi-model inference to identify the most important predictors on SRR rates in response to N fertilisation; predictors include Soil moist and soil temperature (Soil temp), mycorrhizal symbiosis types [AM, ECM, AM+ECM, and non-mycorrhizal (NM)], root respiration measurement method, and N fertiliser amount. To examine potential confounding effects of plant growth forms from that of mycorrhizal association, we compared N fertilisation effects on the variation of SRR between woody and herbaceous plants. We further assessed the effect of N fertilisation on root N concentration for the same studies that also had information on SRR. We hypothesised that N fertilisation would reduce SRR in ECM plants because of reduced reliance on the symbiotic relationship with ECM fungi but that N fertilisation would not affect SRR in plants associated with AM because these plants still rely on the symbiotic relationship with AM fungi for P uptake. Furthermore, given the lack of symbiosis in NM plants, we hypothesise that N fertilisation would have no effect on SRR in NM plants.

## Methods

### Literature Survey and Inclusion Criteria

We collected data related to SRR from published literature studies by searching in the Scopus and ScienceDirect databases in June 2021. We used the advanced search engine and searched for research articles published using the following keywords: (1) “specific root respiration,” (2) “root respiration” AND “root biomass,” (3) “autotrophic respiration” AND “root biomass” OR “fertilisation,” (4) “rhizosphere respiration” AND “root biomass” OR “fertilisation,” and (5) “below-ground respiration” AND “root biomass” OR “fertilisation.” After excluding conference study papers, we found a total of 1,051 study papers. We applied the following criteria to be included in our meta-analysis: studies should have an N fertilisation treatment (without or in combination with other treatments) and should either report SRR directly or report root biomass and root respiration (or autotrophic respiration) allowing us to calculate SRR. We considered three different methods for calculating SRR: (1) based on the measurements of root respiration on excised roots (“root excavation”), (2) based on the difference in respiration between trenched and non-trenched plots (“trenching”), and (3) based on the difference in respiration between planted and unplanted or root-free/bare soil treatments (“unplanted”). For the trenching and unplanted methods, root biomass measurements in non-trenched and planted treatments also needed to be reported to calculate SRR. We excluded publications that were conducted in (1) hydroponic systems, (2) pots filled with sand only, (3) experiments that used oxygen (O_2_) consumption to estimate respiration, and (4) C isotope pulse labelling experiments that were unable to quantify root respiration. Multiple respiration measurements with time were included as separate observations. We found a total of 32 study papers that fit these criteria (Supplementary Table 1) and retrieved a total of 1,600 observations from these study papers (1,559 observations from field experiments and 41 observations from pot experiments, Supplementary Information).

### Data Collection

We used Plot Digitizer software Ver. 2.6.8 to extract the data from figures. We also extracted Soil moist, temperature, and N concentrations in roots when reported. Soil moist units were reported differently in different publications including volumetric, gravimetric, and water-filled pore space (WFPS). We converted all Soil moist data to gravimetric moisture using the bulk density when reported (including five study papers), or otherwise, we used a bulk density of 1.2 g cm^−3^ (another five study papers) (Bache et al., 2008). For each plant species, we collected information on mycorrhizal association type, including AM association, ECM association, AM+ECM (dual association), and non-symbiotic (NM) association based on plant species known to form an association with different types of mycorrhizas (Brundrett, 2009; Cosme et al., 2018; Teste et al., 2020). We considered AM+ECM association for those observations from mixed forests containing species associated with both AM and ECM and where respiration measurements were obtained by the trenching method. We further recorded whether plants were herbaceous or woody. Fertiliser application amount, experimental type (field vs. pot experiment), and method used for respiration measurement were also recorded. The fertiliser application rate was grouped into low ( ≤ 100 kg N ha^−1^) and high fertilisation rates (> 100 kg N ha^−1^).

### Calculation and Statistical Analysis

Data were back-transformed when they were reported in a natural log. For those study papers that reported SE, we calculated the SD (σ) by multiplying the SE with the root square of the sample size (N). In cases where root respiration was calculated based on the difference between the mean total (non-trenched, planted) and heterotrophic (trenched, unplanted) respiration, the SD for root respiration was calculated using the pooled Equation (1) (Kaltenbach, 2012):

(1) $$\begin{array}{r} \sigma_{tr-hr}=\sqrt{\frac{\sigma_{tr}^{2}}{n}+\frac{\sigma_{hr}^{2}}{m}} \end{array}$$

where σ_tr_ and n are the SD and sample size for total respiration, and σ_hr_ and m are the SD and sample size for heterotrophic respiration, respectively.

The Taylor expansion Equation (2) was used to calculate the SD for SRR when it was derived from the mean of root respiration divided by the mean of root biomass (Kendall, 1987):

(2) $$\begin{array}{r} \sigma_{rr/rb}=\sqrt{\frac{\mu_{rr}^{2}}{\mu_{rb}^{2}}\times \left[\frac{\sigma_{rr}^{2}}{\mu_{rr}^{2}}-2\frac{Cov\left(rr,\;rb\right)}{\mu_{rr}\mu_{rb}}+\frac{\sigma_{rb}^{2}}{\mu_{rb}^{2}}\right]} \end{array}$$

where σ_*rr*/*rb*_ is the SD of SRR, μ_*rr*_ and σ_*rr*_ are the mean and SD for root respiration, and μ_*rb*_ and σ_*rb*_ are the mean and SD for root biomass, respectively.

Finally, for those means with no SD reported, the SD based on the coefficient of variance (CV) averaged across all other observations where SDs were reported was calculated.

The effect size of N fertilisation (LnRR) on SRR and root N concentration and their variance of the effect size Var(LnRR) (Borenstein et al., 2009; Lajeunesse, 2011) were calculated using Equations (3) and (4), respectively:

(3) $$\begin{array}{rcl} LnRR & = & \ln\left(\frac{\mu_{t}}{\mu_{c}}\right) \end{array}$$

(4) $$\begin{array}{rcl} Var\left(LnRR\right) & = & \frac{{\left(\sigma_{c}\right)}^{2}}{N_{c}\mu_{c}^{2}}+\frac{{\left(\sigma_{t}\right)}^{2}}{N_{t}\mu_{t}^{2}} \end{array}$$

where μ_*t*_ and μ_*c*_ are the mean of SRR or root N concentration under fertilisation and control treatments, and σ and N are the SDs and sample size of μ_*t*_ and μ_*c*_, respectively. An inverse variance method including two variance components (see below) was used to calculate the weight of individual studies (Veroniki et al., 2016). The restricted maximum likelihood (REML) method was used to estimate the between-study variance τ^2^ (residual heterogeneity), and a Q-profile method was applied to calculate the 95% CIs (Veroniki et al., 2016).

Mixed effect models were used for meta-estimates and meta-regressions to examine the association of categorical and continuous moderators (or predictor as explanatory variables) with the magnitude of the effect size (Terrer et al., 2019; Rubio-Aparicio et al., 2020). We examined how effect sizes differed among categorical factors of Myco type (AM, ECM, AM+ECM, and NM, both at fertilisation levels ≤ 100 and > 100 kg N ha^−1^), experimental type (field and pot experiments), method (root excavation, trenching, and unplanted), and plant growth form (herbaceous and woody), and continuous factors of Soil moist, Soil temp, and N fertilisation level. Effect sizes and their corresponding variances were calculated in R using the function “*escalc*” from the package *metafor* (Viechtbauer, 2010). Because the corresponding effect size estimates for studies with multiple observations (multiple measurements over time) are likely to be correlated (Gleser and Olkin, 2009; Lajeunesse, 2011; Nakagawa and Santos, 2012), we generated a variance-covariance (VCV) matrix and conducted a multi-level mixed effect model that accounts for the non-independency among effect sizes (Noble et al., 2017; Midolo et al., 2019). We used the function “*rma.mv*” from the package *metafor* where observation ID was nested within the study as a random effect (second variance σ^2^ component), and a VCV matrix was used for weighing the effect sizes (Noble et al., 2017; Midolo et al., 2019; Terrer et al., 2021).

A multi-model inference was further conducted by modelling all possible predictor (moderator) combinations to examine which predictor provided the best fit with N fertilisation effect sizes on SRR, based on the sum of Akaike weights (AICc) and *p* > 0.05 (Burnham, 2002; Gurka, 2006). We further conducted a meta-regression relating LnRR of SRR to N fertilisation with root N concentration in fertilised treatments.

All analyses were done in R, version 4.0.0 (R Development Core Team, 2020). We used the package *metafor* to conduct the multi-level meta-analysis and meta-regressions (Viechtbauer, 2010) and *glmulti* for model selection and multi-model inference (Calcagno and de Mazancourt, 2010).

## Results

**Figure 1:**
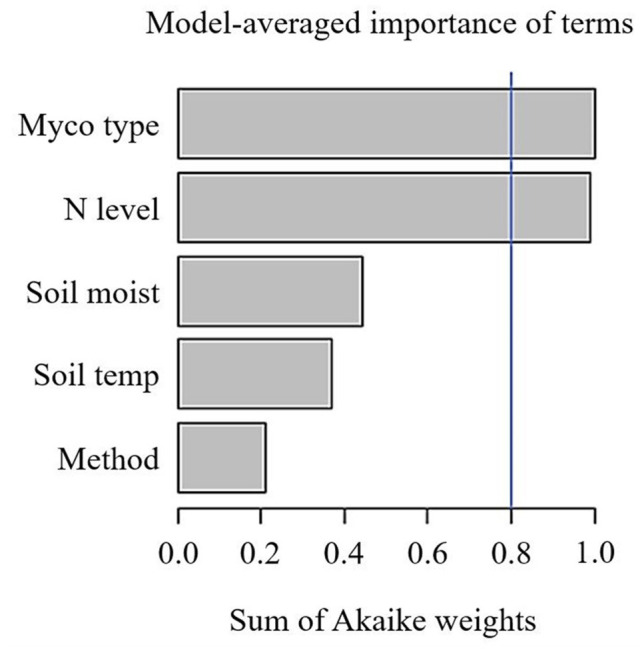
Sum of Akaike weights (AICc) from the multi-model inference for predictors including mycorrhizal type (Myco type), N fertilisation levels (N level), soil temperature (Soil temp), gravimetric soil moisture (Soil moist), Specific root respiration measurement method on N fertilisation effect sizes of specific root respiration. Cut-off value (blue line) is set at 0.8 differentiating between important and less important predictors. N.B. only observations from field experiments are included.

Across 1,600 observations from 32 publications, SRR was not affected by N fertilisation in field or pot experiments (Table 1). Because the majority of observations came from field experiments (1,559 observations), the remainder of our analysis is focused on these studies. Given the large variation in effect sizes among observations, we conducted a multi-model inference to find out the most important moderators on effect sizes of SRR. We included Myco type, Soil moist, Soil temp, N fertiliser level ( ≤ 100 kg N ha^−1^ and > 100 kg N ha^−1^), and measurement method. Results of our model selection showed that Myco type and N fertiliser level were the two most important moderators explaining variation in N fertilisation effect sizes, with both moderators showing a sum of AICc larger than the cut-off value 0.8 (Figure 1).

**Table 1:** Effect sizes (LnRR) of specific root respiration (SRR) and root N concentration to N fertilisation for all observations and separated by field and pot experiments.

	***n***	**Estimate**	**Lower CI**	**Upper CI**	***p***	**Model p**
**Specific root respiration**	1,600	0.059	−0.049	0.181	0.3	
Field experiments	1,559	0.041	−0.079	0.162	0.5	0.3
Pot experiments	41	0.240	−0.087	0.567	0.2	
**Root N concentration**	258	0.200	−0.006	0.407	0.06	
Field experiments	234	0.247	0.012	0.482	0.03	0.1
Pot experiments	24	−0.012	−0.549	0.512	0.9	

*Estimates for LnRR are associated with lower and upper 95% CIs. Significant p-values (p < 0.05) are shown in bold (n is the number of observations)*.

We found no significant variation for SRR to N fertilisation among different respiration measurement methods (Table 2) and not between woody and herbaceous plants (Table 3).

**Table 2:** Effect sizes (LnRR) of SRR to N fertilisation for different respiration measurement methods.

**Measurement method**	***n***	**Estimate**	**Lower CI**	**Upper CI**	***p*-value**	**Model p**
Root excavation	26	0.073	−0.387	0.533	0.7	0.5
Trenching	1,268	0.026	−0.119	0.171	0.7	
Unplanted	306	0.173	−0.065	0.412	0.1	

*Estimates for LnRR are associated with lower and upper 95% CIs. Significant p-values (p < 0.05) are shown in bold (n is the number of observations)*.

**Table 3:** Effect sizes (LnRR) of SRR to N fertilisation for different plant growth forms.

**Plant growth form**	***n***	**Estimate**	**Lower CI**	**Upper CI**	***p*-value**	**Model p**
Herbaceous plants	874	0.052	−0.150	0.254	0.6	0.8
Woody plants	685	0.035	−0.136	0.205	0.7	

*Estimates for LnRR are associated with lower and upper 95% CIs. Significant p-values (p < 0.05) are shown in bold (n is the number of observations, and only observations from field experiments are included)*.

**Figure 2:**
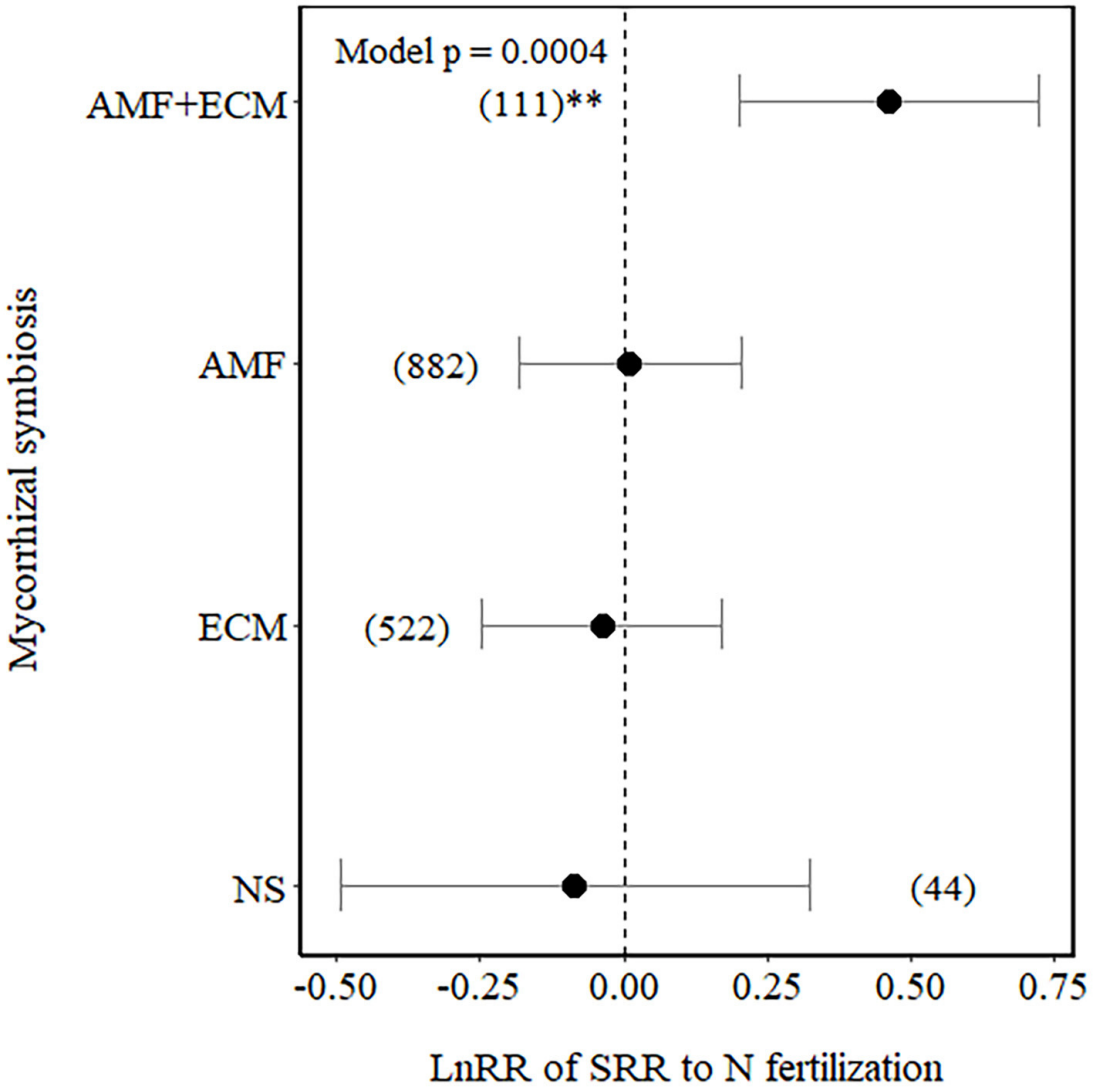
Effect sizes (LnRR) of specific root respiration (SRR) to N fertilisation in plants with no mycorrhizal associations (NM), with ectomycorrhizal associations (ECM) only, arbuscular mycorrhizal associations (AM) only, or AM+ECM associations. Numbers in brackets represent the number of observations, and error bars represent the 95% CIs. **indicates significance at *p* < 0.01. Only observations from field experiments are included.

Among the Myco types, SRR increased in plants with dual association (AM+ECM) but was not affected in plants associated with AM only, ECM only, and NM plants only (NM), although NM plants showed a higher variation in the effect size compared to the other Myco types (Figure 2).

**Figure 3:**
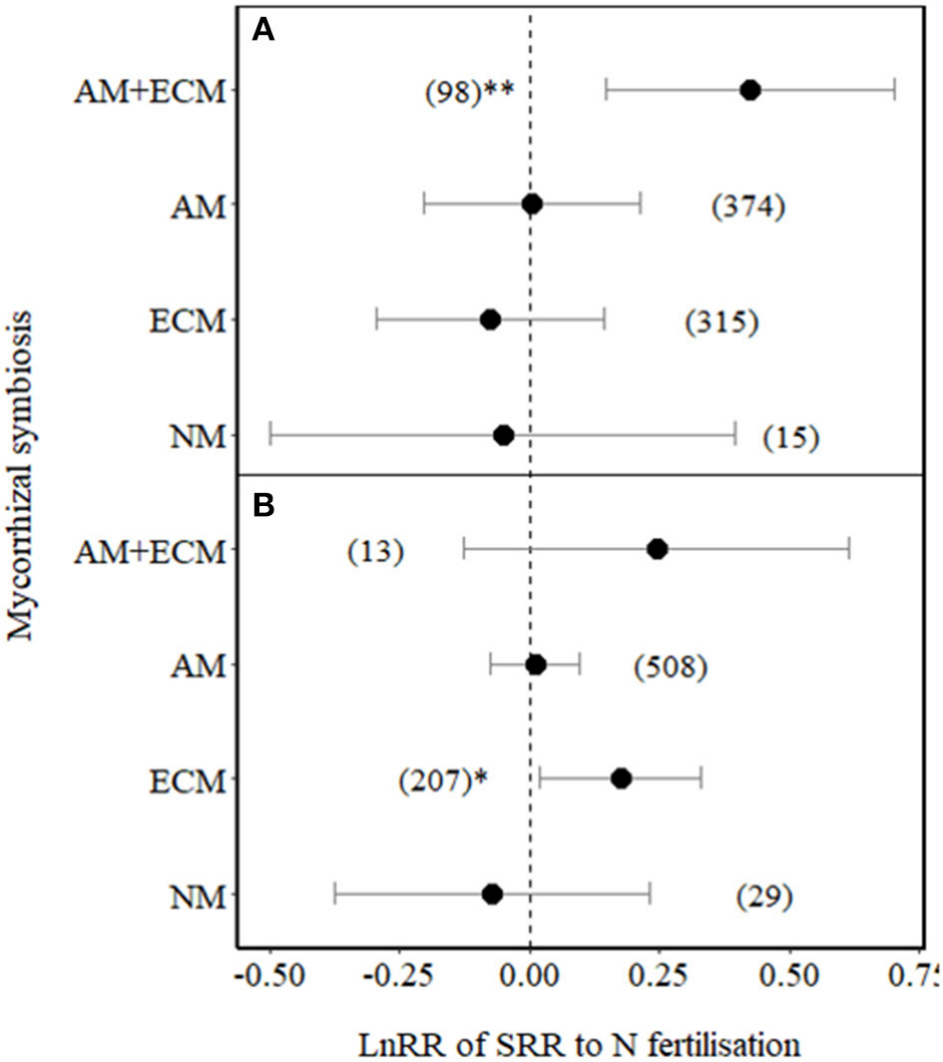
Effect sizes (LnRR) of SRR in plants with no mycorrhizal associations (NM), with ECM associations only, AM associations only, or AM+ECM associations, for **(A)** low N fertilisation levels (*N* ≤ 100 kg ha^−1^) and **(B)** high N fertilisation levels (*N* > 100 kg ha^−1^). Numbers in brackets represent the number of observations and error bars represent the 95% CIs. ** and * indicate significance at *p* < 0.01 and *p* < 0.05, respectively. Only observations from field experiments are included.

We then tested whether the effect size of SRR in plants associated with different types of mycorrhizas varied between low ( ≤ 100 kg N ha^−1^) and high N (> 100 kg N ha^−1^) fertilisation levels (Figure 3). SRR significantly increased in plants with AM+ECM under low N fertilisation (*p* = 0.01, Figure 3A). Under high N fertilisation, SRR increased in ECM plants (Figure 3B), while it showed a non-significant increase in plants associated with AM+ECM (but note the low number of observations for AM+ECM association with high variation in effect sizes in response to high N fertilisation). SRRs in AM and NM plants were not affected by either low or high N fertilisation (also note the relative low number of observations and high variation in effect sizes in response to both low and high N fertilisation for NM plants, Figure 3).

**Figure 4:**
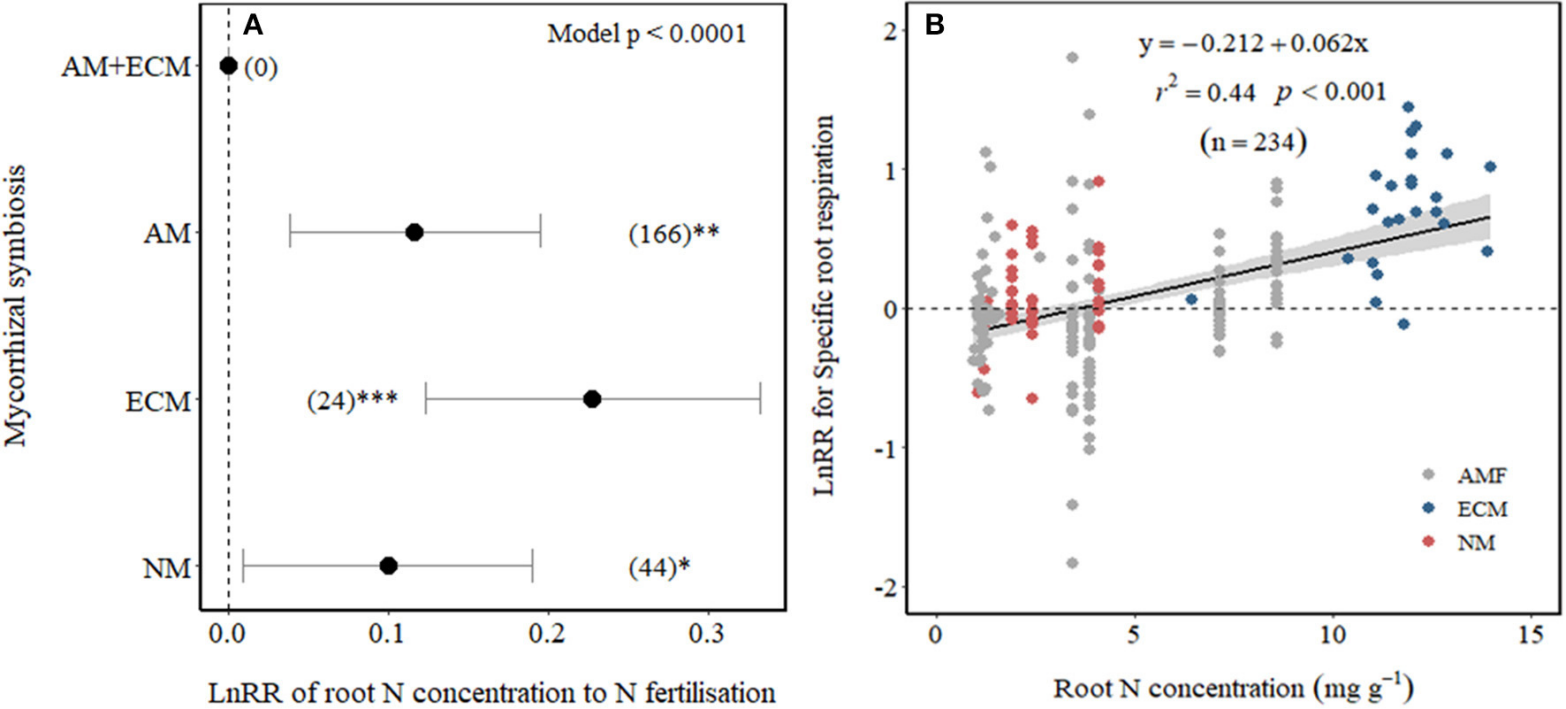
Effect sizes (LnRR) of root N concentration to N fertilisation **(A)** in association with different mycorrhizal symbiosis and **(B)** meta-regression relating LnRR of SRR to N fertilisation with root N concentration in fertilised treatments (NM, non-mycorrhizal—red symbols; ECM, ectomycorrhizal—blue symbols; and AM, arbuscular mycorrhizal—grey symbols). ***, **, and *indicate significance at *p* < 0.0001, *p* < 0.01, and *p* < 0.05, respectively. Shade band around the regression fit represents 95% CIs. Only observations from field experiments are included.

We examined whether differences among Myco type in SRR effect sizes to N fertilisation could be explained by their differences in root N concentration. As expected, root N concentration increased with N fertilisation across all Myco types in field experiments (*p* = 0.03) but not in pot experiments (Table 1). Plants associated with ECM showed the largest increase in root N concentration with N fertilisation and with smaller increases in root N concentration of AM and NM plants (Figure 4A), while there were no observations for AM+ECM plants. Due to the low number of observations, we were unable to test whether there were differences in the effect sizes of root N concentration between low and high N fertilisation levels. A meta-regression relating SRR affected by N fertilisation to root N concentration in fertilised treatments revealed a positive relationship explaining 44% of the variation (*p* < 0.001, *r*^2^ = 0.44, Figure 4B).

## Discussion

The mycorrhizal type was the most important predictor of N fertilisation effect sizes on SRR, with no changes for AM, ECM, and NM plants and no increases for AM+ECM plants. N fertilisation level was the second most important moderator of SRR effect sizes. In particular, when both factors were examined together, SRR increased in ECM plants at high N fertilisation rates (>100 kg N ha^−1^) and for AM+ECM plants at low N fertilisation rates ( ≤ 100 kg N ha^−1^). While N fertilisation effects on SRR can be due to several factors, such as fertiliser-induced changes in root architecture and morphology (López-Bucio et al., 2003), root N concentrations (Reich et al., 2008), and mycorrhizal colonisation (Nilsson and Wallander, 2003; Treseder, 2004; Emmanuel et al., 2012), in this study, we focus on explaining these effects from a C cost and N benefit perspective of mycorrhizal symbiosis (Phillips et al., 2013).

Measurement method did not play an important role on SRR (Table 2), despite the fact that respiration measured by “trenching” and “unplanted” methods would also include external mycelial respiration (and possibly decomposition of rhizodeposition), while the “root excavation” method would only include internal mycelial respiration in SRR. This suggests that external mycelial respiration and decomposition of rhizodeposition were little affected by N fertilisation (Nilsson and Wallander, 2003; Hobbie, 2008; Wang et al., 2021). Further, we found no variation in SRR to N fertilisation between woody and herbaceous plants (Table 3), indicating that our results were not confounded by plant growth form. Interestingly, Soil temp and moist did not contribute significantly to explaining the variance in SRR effect sizes. Although Soil temp and moisture have been shown as effective factors controlling SRR (Atkin et al., 2000; Zhou et al., 2016), our results highlight that N fertilisation plays a more important role than Soil temp and moist on root respiration (Chen et al., 2019).

From the C cost and N benefit perspective, increased N availability (i.e., with increased N fertilisation) can decrease plant C cost as a result of direct N uptake so that mycorrhizal symbiosis may become too C expensive. A decrease in C cost may particularly occur in ECM plants. For instance, the excretion of extracellular enzymes by ECM fungi to decompose organic matter and release N for plant use (Pritsch and Garbaye, 2011; Nicolás et al., 2019) is C expensive, and it may be more C cost-effective for those plants to take up N directly from the soil when the availability of N increases. Furthermore, the extraradical mycelial networks that ECM fungi build to forage for nutrients in nutrient-poor soils (Chen et al., 2019) require a substantial amount of plant C (Soudzilovskaia et al., 2015) and may not be a C-efficient way for plants to acquire nutrients. Consequently, a reduction in SRR, due to reduced formation of mycorrhizal associations with increased N availability (Nilsson and Wallander, 2003; Treseder, 2004; Emmanuel et al., 2012), is expected as a plant C cost management strategy for ECM plants. To our surprise, we did not observe this but, instead, observed an increase in SRR in ECM plants at high levels of N fertilisation.

**Figure 5:**
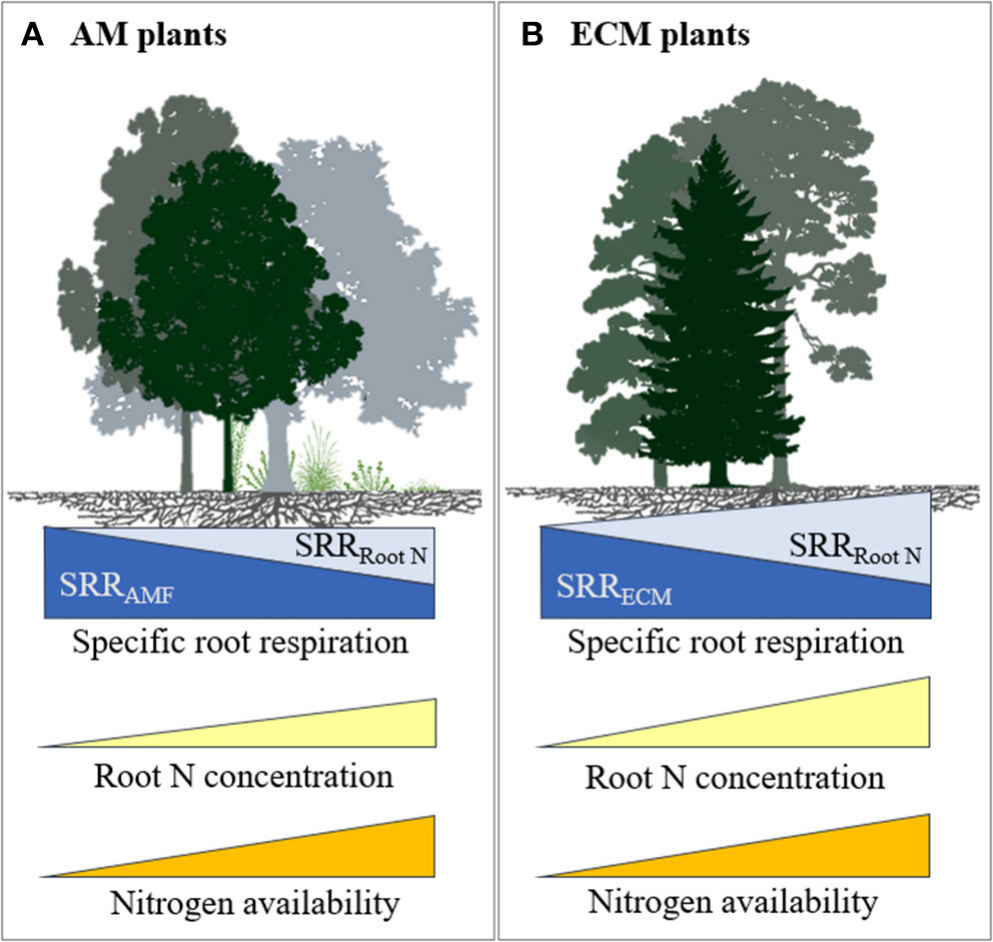
Conceptual model illustrating the influence of soil N availability on SRR and root N concentration for plants in association with **(A)** AM and **(B)** ECM fungi. With increased soil N availability, both ECM and AM plants spend less C to support mycorrhiza resulting in reduced SRR associated with mycorrhizal symbiosis (a dark blue component of SRR) but respire more C due to an increase in root N concentration (a light blue component of SRR). However, the increase in plants C costs associated with higher root N concentration outweighs the reduction in C to support ECM fungi, while it counterbalances the reduction in C to support AM fungi. Furthermore, in AM plants, C costs for mycorrhizal symbiosis may remain relatively high at high soil N availability, because AM symbiosis is more important for P uptake. We refrained from drawing models for NM and AM+ECM plants due to limited data.

There are several possibilities that could explain our results. For instance, high rates of N fertilisation may not have decreased mycorrhizal colonisation and hyphal growth of ECM fungi and associated C cost. Although this is not supported by some studies in ECM trees where N fertilisation caused a decrease in mycelial production (Nilsson and Wallander, 2003; Ekblad et al., 2016), in a meta-analysis Treseder (2004) observed no significant effect of N fertilisation on ECM colonisation. It is also possible that N fertilisation may have changed the mycorrhizal communities (Avis et al., 2003; Parrent et al., 2006) towards species that require more C from their plant host, thereby increasing SRR. It is further possible that N fertilisation increased SRR in ECM plants because of an associated increase in root N concentration. Our results showed that when plants respired more C per unit of root biomass, they also took up more N with increased N fertilisation. The positive relationship between root N concentration and SRR suggests that higher N uptake results in greater C cost, possibly as a result of increased metabolic activities related to nutrient uptake, assimilation, and transportation (Reich et al., 2008; Burton et al., 2012). Therefore, any reductions in C costs due to lower mycorrhizal colonisation may have been outweighed by the increased C costs associated with the higher root N concentrations through increased direct uptake, which resulted in increased SRR at high N fertilisation rates (>100 kg N ha^−1^) (see conceptual diagram in Figure 5).

Unlike ECM, our results showed that N fertilisation did not affect SRR in AM plants, under low or high N (Figure 3). One possible explanation is that AM colonisation is less affected by N availability and is more important for P supply so that the fungal contribution to SRR may not be affected by N availability (Hughes et al., 2008). However, AM colonisation on average decreased with N fertilisation by 15% in a meta-analysis (Treseder, 2004), although there was large variation among studies, possibly due to large variation in available N in control treatments. An alternative explanation may be that a reduction in mycorrhizal colonisation with N fertilisation may have been counterbalanced by an increase in root N concentration on SRR (Figure 5). Therefore, in the case of AM plants, reduced C costs *via* reduced AM colonisation may have been offset by the increased C costs associated with greater plant N uptake (Reich et al., 2008), resulting in no net change in SRR in response to N fertilisation.

It might be expected that SRR declines with N fertilisation in plants with the dual mycorrhizal association (AM+ECM) because supporting both types of mycorrhizal association could be more C expensive for plants than supporting one type. However, our results showed that SRR increased with N fertilisation (significant at N ≤ 100 kg ha^−1^) in plants with the dual association (Figure 3A). Considering the different capabilities of AM and ECM fungi to acquire nutrients from organic and inorganic sources, they may play a complementary role in N and P uptake (Teste et al., 2020). Increased inorganic N availability with N fertilisation could shift the dominance of mycorrhizal association from ECM acquiring organic forms of N to AM fungi acquiring inorganic N (Phillips et al., 2013), without causing a reduction in mycorrhizal colonisation, but where fertilisation-induced enhancement in root N concentrations would increase respiratory C cost. N fertilisation can also induce a shift from N to P limitation in plants, which could increase plant C cost, given that soil P availability is usually much lower and less mobile compared to N.

Specific root respiration in NM plants was not affected by N fertilisation. If the effects of N fertilisation on SRR are controlled by mycorrhizal associations, then this would be expected. Conversely, if N fertilisation also increased root N concentration, then this would increase SRR. However, the large variation in the effect size of SRR in NM plants among studies precludes making any generalisations. NM plants can be categorised into early colonisers that have no morphological root adaptations and thrive in disturbed or relatively fertile soils, and plants with strong morphological root adaptations, such as cluster or dauciform roots that help plants with P acquisition in severely P-impoverished soils (Lambers and Teste, 2013). These two groups grow in soils that contrast in nutrient availability, and this could explain the large variation in N responses for NM plants, although we did not have enough observations to examine this for low and high N fertilisation levels.

We found that SRR increased in ECM plants supplied with more than 100 kg N ha^−1^ but not in AM and NM plants, while root N concentrations also increased the most in ECM plants in response to N fertilisation. These results provide insights into why ECM plants tend to dominate in organic or high C:N ratio soils and AM plants in mineral or low C:N ratio soils (Taylor et al., 2016; Jo et al., 2019; Soudzilovskaia et al., 2019). N acquisition through ECM association seems less C expensive for plants when soil N availability is low. This frequently occurs in high C:N ratio soils where most of the soil N is in organic form. Under these conditions, ECM plants may have an advantage over AM plants in acquiring N from organic sources *via* excretion of extracellular enzymes (Pritsch and Garbaye, 2011; Sulman et al., 2017). With increased inorganic N availability caused by N fertilisation, root N concentrations increase, and at some point this may become too C expensive and no longer economical for plants to support ECM associations. In contrast, supporting AM associations when soil N availability is high may still be beneficial to plants. Although C costs could increase because of an increase in root N concentration, other nutrients like P may become more limiting to plant growth and AM symbiosis may be advantageous (Jo et al., 2019), given that AM fungi play an important role in P acquisition (van der Heijden et al., 2015) and are believed to be less C costly for plants than the ECM symbiosis (Viertelhauzen, 2013). SRR may, therefore, be a key variable in explaining responses of plant growth and their dependence on mycorrhizal associations with N fertilisation and increased atmospheric N deposition. Our meta-analysis further highlights the need for future research studies addressing mechanisms (e.g., biochemical and metabolic pathways) causing variation in SRR and plant N uptake associated with different Myco types in response to N fertilisation.

## Data Availability Statement

The original contributions presented in the study are included in the article/Supplementary Material, further inquiries can be directed to the corresponding author/s.

## Author Contributions

FD conceived the research idea and participated in the evaluation of the results. BB collected the data, conducted the meta-analysis, performed the model-selection and multi-model inference, and drafted the manuscript. All authors contributed to editing the manuscript.

## Conflict of Interest

The authors declare that the research was conducted in the absence of any commercial or financial relationships that could be construed as a potential conflict of interest.

## Publisher's Note

All claims expressed in this article are solely those of the authors and do not necessarily represent those of their affiliated organizations, or those of the publisher, the editors and the reviewers. Any product that may be evaluated in this article, or claim that may be made by its manufacturer, is not guaranteed or endorsed by the publisher.
