# Supplementary material for: Nitrogen Fertilisation Increases Specific Root Respiration in Ectomycorrhizal but Not in Arbuscular Mycorrhizal Plants: A Meta-Analysis
Source: Front Plant Sci. 2021 Aug 6;12:711720. doi: 10.3389/fpls.2021.711720 (PMC8377726; doi:10.3389/fpls.2021.711720)
Supplement: Supplementary file 1 [file Table_1.docx]

***Supplementary file***

Table S1. List of papers which was used for data extraction with main information including experiment type, plant name, mycorrhizal type, ecosystem and respiration measurement method.

| **Row** | **Reference** | **Exp. type** | **Plant name** | **Mycorrhizal association** | **Ecosystem** | **Respiration method** |
| --- | --- | --- | --- | --- | --- | --- |
| 1 | Burton et al. (2004) | Field | *Acer saccharum* Marsh. | AM | Forest | Root excavation |
| 2 | Chen et al. (2016) | Field | Mixture | AM | Grassland | Trenching |
|  |  |  | *Carex korshinskii* | NM | Grassland | Trenching |
|  |  |  | *Leymus chinensis* | AM | Grassland | Trenching |
|  |  |  | *Agropyron cristatum* | AM | Grassland | Trenching |
|  |  |  | *Achnatherum sibiricum* | AM | Grassland | Trenching |
|  |  |  | *Stipa grandis* | AM | Grassland | Trenching |
| 3 | Chen et al. (2019) | Field | *Tilia amurensis*, *Pinus koraiensis* and *Quercus mongolica* | AM+ECM | Forest | Trenching |
| 4 | Comeau et al. (2016) | Field | *Elaeis guineensis* | AM | Forest | Trenching |
| 5 | Ding et al. (2010) | Field | *Zea mays* L. | AM | Crop | Unplanted |
| 6 | Drake et al. (2012) | Field | *Pinus taeda* | ECM | Forest | Trenching |
| 7 | Gavrichkova and Kuzyakov (2008) | Pot | *Lupinus albus* | AM | Crop | Unplanted |
|  |  |  | *Zea mays* L. | AM | Crop | Unplanted |
| 8 | Li et al. (2018) | Field | *Quercus acutissima* and *Q. variabilis* as dominant species | ECM | Forest | Trenching |
| 9 | Li et al. (2019) | Field | *Elymus nutans, Poa crymophila And Kobresia humilis* | AM | Grassland | Trenching |
| 10 | Liang et al. (2019) | Field | deciduous oak mixed forest | ECM | Forest | Trenching |
| 11 | Liu et al. (2019) | Field | *Castanopsis fargesii*, *Lithocarpus glaber* and *Schima superba* | AM+ECM | Forest | Unplanted |
| 12 | Lovelock et al. (2006) | Field | *Rhizophora mangle* was dominated tree | AM | Forest | Root excavation |
| 13 | Lu et al. (2018) | Pot | *Leymus chinensis* (Trin.) Tzvel. | AM | Crop | Unplanted |
|  |  | Pot | *Medicago sativa* L. | AM | Crop | Unplanted |
| 14 | Lu et al. (1998) | Pot | *Pseudotsuga menziesii* (Mirb.) Franco | ECM | Forest | Unplanted |
| 15 | Morell et al. (2012) | Field | *Hordeum vulgare* L., cv. Hispanic | AM | Crop | Unplanted |
| 16 | Peng et al. (2017) | Field | *Stipa purpurea*, *Carex rigescens*, *Poa crymophila* Keng, *Agropyron cristatum*, *Heteropappus altaicus* (Willd.) Novopokr as dominated species | AM | Grassland | Trenching |
| 17 | Sun et al. (2013) | Field | *Triticum aestivum* L. cv. Yangmai 14 | AM | Crop | Trenching |
| 18 | Sun et al. (2014) | Field | *L. principis-rupprechtii* | ECM | Forest | Trenching |
| 19 | Tu et al. (2013) | Field | *Pleioblastus amarus* | NM | Forest | Unplanted |
| 20 | Wang and liu (2014) | Pot | *Pinus tabulaeformis* | ECM | Forest | Root excavation |
| 21 | Wang et al. (2019) | Field | *Picea baifouriana*, *Populus* spp. | AM+ECM | Forest | Trenching |
|  |  | Field | *Abies georgei*, *Picea likiangensis* as dominated trees | ECM | Forest | Trenching |
| 22 | Wang et al. (2017) | Field | *alpine spruces* | ECM | Forest | Unplanted |
| 23 | Wei et al. (2018) | Field | *Stipa bungeana*, *S. grandis*, *S. przewalskyi* as dominant plants | AM | Grassland | Trenching |
| 24 | Zeng et al. (2018a) | Field | *Leymus chinensis*, *Stipa baicalensis* and *S. grandis* | AM | Grassland | Trenching |
| 25 | Zeng et al. (2018b) | Field | evergreen Pinus *sylvestris var. mongolica* (Mongolia pine, 85%) and deciduous *Larix principis-rupprechtii* (birch, 15%) | ECM | Forest | Trenching |
| 26 | Zeng and Wang (2015) | Field | evergreen *Pinus sylvestris var. mongolica* (Mongolia pine, 85%) and deciduous *Larix principis-rupprechtii* (birch, 15%) | ECM | Forest | Trenching |
| 27 | Zeng et al. (2020) | Field | *Pinus sylvestris* | ECM | Forest | Trenching |
| 28 | Zhang et al. (2014) | Field | *Stipa bungeana, Artemisia frigida* and *Tripolium vulgare* Ness | AM | Grassland | Trenching |
| 29 | Zhang et al. (2021) | Field | *Quercus acutissima* and *Q. variabilis* | AM+ECM | Forest | Trenching |
| 30 | Zhao et al. (2018) | Field | *Pinus tabulaeformis* as dominant | ECM | Forest | Trenching |
| 31 | Zhong et al. (2016) | Field | *Triticum aestivum* L. cv., Changhan No. 58 | AM | Crop | Trenching |
| 32 | Zhu et al. (2018) | Pot | *Oryza sativa* L. ‘Two-line hybrid rice Zhongzao 39’ | AM | Crop | Unplanted |

**References:**

Burton, A.J., Pregitzer, K.S., Crawford, J.N., Zogg, G.P., Zak, D.R. (2004). Simulated chronic NO_3_^−^ deposition reduces soil respiration in northern hardwood forests. Glob Chang Biol. 10(7), 1080-1091.

Chen, D., Li, J., Lan, Z., Hu, S., Bai, Y. (2016). Soil acidification exerts a greater control on soil respiration than soil nitrogen availability in grasslands subjected to long-term nitrogen enrichment. Func Ecol. 30(4), 658-669.

Chen, F., Yan, G., Xing, Y., Zhang, J., Wang, Q., Wang, H. et al. (2019). Effects of N addition and precipitation reduction on soil respiration and its components in a temperate forest. Agric For Meteorol. 271, 336-345.

Comeau, L.-P., Hergoualc'h, K., Hartill, J., Smith, J., Verchot, L.V., Peak, D. et al. (2016). How do the heterotrophic and the total soil respiration of an oil palm plantation on peat respond to nitrogen fertilizer application? Geoderma. 268, 41-51.

Ding, W., Yu, H., Cai, Z., Han, F., Xu, Z. (2010). Responses of soil respiration to N fertilization in a loamy soil under maize cultivation. Geoderma. 155(3), 381-389.

Drake, J.E., Oishi, A.C., Giasson, M.A., Oren, R., Johnsen, K.H., Finzi, A.C. (2012). Trenching reduces soil heterotrophic activity in a loblolly pine (*Pinus taeda*) forest exposed to elevated atmospheric CO_2_ and N fertilization. Agric For Meteorol. 165, 43-52.

Gavrichkova, O., Kuzyakov, Y. (2008). Ammonium versus nitrate nutrition of Zea mays and Lupinus albus: Effect on root-derived CO_2_ efflux. Soil Biol Biochem. 40(11), 2835-2842.

Li, W., Wang, J., Li, X., Wang, S., Liu, W., Shi, S. et al. (2019). Nitrogen fertilizer regulates soil respiration by altering the organic carbon storage in root and topsoil in alpine meadow of the north-eastern Qinghai-Tibet Plateau. Scientific Reports. 9(1), 13735.

Li, Y., Sun, J., Tian, D., Wang, J., Ha, D., Qu, Y. et al. (2018). Soil acid cations induced reduction in soil respiration under nitrogen enrichment and soil acidification. Sci Total Environ. 615, 1535-1546.

Liang, L., Chen, F., Han, H., Zhang, Y., Zhu, J., Niu, S. (2019). Pathways regulating decreased soil respiration with nitrogen addition in a subtropical forest in China. Water, Air, & Soil Pollution. 230(4), 91.

Liu, H., Zhou, G., Bai, S.H., Song, J., Shang, Y., He, M. et al. (2019). Differential response of soil respiration to nitrogen and phosphorus addition in a highly phosphorus-limited subtropical forest, China. Forest Ecology and Management. 448, 499-508.

Lovelock, C.E., Ruess, R.W., Feller, I.C. (2006). Fine root respiration in the mangrove Rhizophora mangle over variation in forest stature and nutrient availability. Tree Physiol. 26(12), 1601-1606.

Lu, J., Dijkstra, F.A., Wang, P., Cheng, W. (2018). Rhizosphere priming of grassland species under different water and nitrogen conditions: a mechanistic hypothesis of C-N interactions. Plant Soil. 429(1), 303-319.

Lu, S., Mattson, K.G., Zaerr, J.B., Marshall, J.D. (1998). Root respiration of Douglas-fir seedlings: effects of N concentration. Soil Biol Biochem. 30(3), 331-336.

Morell, F.J., Whitmore, A.P., Álvaro-Fuentes, J., Lampurlanés, J., Cantero-Martínez, C. (2012). Root respiration of barley in a semiarid Mediterranean agroecosystem: field and modelling approaches. Plant Soil. 351(1), 135-147.

Peng, Y., Li, F., Zhou, G., Fang, K., Zhang, D., Li, C. et al. (2017). Nonlinear response of soil respiration to increasing nitrogen additions in a Tibetan alpine steppe. Environ Res Lett. 12(2), 024018.

Sun, H.-F., Zhu, J.-G., Xie, Z.-B., Liu, G., Tang, H.-Y. (2013). Effect of Atmospheric CO_2_ Enrichment on Soil Respiration in Winter Wheat Growing Seasons of a Rice-Wheat Rotation System. Pedosphere. 23(6), 752-766.

Sun, Z., Liu, L., Ma, Y., Yin, G., Zhao, C., Zhang, Y. et al. (2014). The effect of nitrogen addition on soil respiration from a nitrogen-limited forest soil. Agric For Meteorol. 197, 103-110.

Tu, L.-h., Hu, T.-x., Zhang, J., Li, X.-w., Hu, H.-l., Liu, L. et al. (2013). Nitrogen addition stimulates different components of soil respiration in a subtropical bamboo ecosystem. Soil Biol Biochem. 58, 255-264.

Wang, G., liu, F. (2014). Carbon allocation of Chinese pine seedlings along a nitrogen addition gradient. For Ecol Manag. 334, 114-121.

Wang, J., Wang, G., Fu, Y., Chen, X., Song, X. (2019). Short-term effects of nitrogen deposition on soil respiration components in two alpine coniferous forests, southeastern Tibetan Plateau. J For Res. 30(3), 1029-1041.

Wang, J., Wang, G., Zhaoyong, H. (2017). Short-term effect of nitrogen addition on microbial and root respiration in an alpine spruce ecosystem. Int J Bioautomation. 21, 145-159.

Wei, L., Su, J., Jing, G., Zhao, J., Liu, J., Cheng, J. et al. (2018). Nitrogen addition decreased soil respiration and its components in a long-term fenced grassland on the Loess Plateau. J Arid Environ. 152, 37-44.

Zeng, W., Chen, J., Liu, H., Wang, W. (2018a). Soil respiration and its autotrophic and heterotrophic components in response to nitrogen addition among different degraded temperate grasslands. Soil Biol Biochem. 124, 255-265.

Zeng, W., Wang, W. (2015). Combination of nitrogen and phosphorus fertilization enhance ecosystem carbon sequestration in a nitrogen-limited temperate plantation of Northern China. For Ecol Manag. 341, 59-66.

Zeng, W., Zhang, J., Dong, L., Wang, W., Zeng, H. (2020). Nonlinear responses of total belowground carbon flux and its components to increased nitrogen availability in temperate forests. Science of The Total Environment. 715, 136954.

Zeng, W., Zhang, J., Wang, W. (2018b). Strong root respiration response to nitrogen and phosphorus addition in nitrogen-limited temperate forests. Sci Total Environ. 642, 646-655.

Zhang, C., Niu, D., Hall, S.J., Wen, H., Li, X., Fu, H. et al. (2014). Effects of simulated nitrogen deposition on soil respiration components and their temperature sensitivities in a semiarid grassland. Soil Biol Biochem. 75, 113-123.

Zhang, J., Li, Y., Wang, J., Chen, W., Tian, D., Niu, S. (2021). Different responses of soil respiration and its components to nitrogen and phosphorus addition in a subtropical secondary forest. Forest Ecosystems. 8(1), 37.

Zhao, B., Geng, Y., Cao, J., Yang, L., Zhao, X. (2018). Contrasting Responses of Soil Respiration Components in Response to Five-Year Nitrogen Addition in a Pinus tabulaeformis Forest in Northern China. Forests. 9(544).

Zhong, Y., Yan, W., Zong, Y., Shangguan, Z. (2016). Biotic and abiotic controls on the diel and seasonal variation in soil respiration and its components in a wheat field under long-term nitrogen fertilization. Field Crops Res. 199, 1-9.

Zhu, Z., Ge, T., Liu, S., Hu, Y., Ye, R., Xiao, M. et al. (2018). Rice rhizodeposits affect organic matter priming in paddy soil: The role of N fertilization and plant growth for enzyme activities, CO_2_ and CH_4_ emissions. Soil Biol Biochem. 116, 369-377.
